# Evaluation of Carotid Stenosis in a High‐Stroke‐Risk Population by Hemodynamic Dual‐Parameters Based on Ultrasound Vector Flow Imaging

**DOI:** 10.1002/brb3.70150

**Published:** 2024-11-17

**Authors:** Shaofu Hong, Yinghui Dong, Wenjing Gao, Di Song, Mengmeng Liu, Weiyue Li, Yigang Du, Jinfeng Xu, Fajin Dong

**Affiliations:** ^1^ Department of Ultrasound Shenzhen People's Hospital (The Second Clinical Medical College, Jinan University; The First Affiliated Hospital, Southern University of Science and Technology) Shenzhen Guangdong China; ^2^ Department of Radiology Shenzhen People's Hospital (The Second Clinical Medical College, Jinan University; The First Affiliated Hospital, Southern University of Science and Technology) Shenzhen Guangdong China; ^3^ Ultrasound R&D Department Shenzhen Mindray Bio‐Medical Electronics Co., Ltd. Shenzhen Guangdong China

**Keywords:** carotid artery stenosis, stroke, turbulence, vector flow imaging, wall shear stress

## Abstract

**Objective:**

This study explored the feasibility of using high‐frame‐rate ultrasound vector flow imaging (VFI) to quantitatively assess hemodynamics in atherosclerotic internal carotid artery stenosis (ICAS) by evaluating dual‐parameters, turbulence index (Tur), and wall shear stress (WSS). Their efficacy in evaluating carotid artery stenosis was also analyzed.

**Methods:**

Fifty‐nine patients with ICAS were enrolled. B‐mode ultrasound and V Flow (a high‐frame‐rate VFI) were performed using the Resona R9 system. The stenosis rate was measured in grayscale mode, whereas the time‐averaged Tur index, the time‐averaged WSS (WSS_mean_), and maximum WSS (WSS_max_) around stenosis were measured. The combined diagnostic efficacy of Tur inand WSS was also investigated.

**Results:**

Compared to proximal to stenosis (Tur index, 2.88% ± 3.65%), highly disordered blood flow was observed in the stenotic (23.17% ± 15.52%, *p* < 0.001) and distal segment (25.86% ± 17.29%, *p* < 0.001). WSS_max_ ([11.91 ± 6.73] vs. [4.43 ± 5.4] Pa, *p* < 0.001) and WSS_mean_ ([3.42 ± 2.67] vs. [0.86 ± 1.21] Pa, *p* < 0.001) were significantly bigger in stenotic than those in the distal segment. The differences in the ratio WSS_max_/Tur or WSS_mean_/Tur among different segments around stenosis were statistically significant (*p* < 0.001). The combination of Tur index and WSS had the best diagnostic performance in ICAS (AUC, 0.899).

**Conclusion:**

The application of Tur index and WSS for quantitative assessment of ICAS hemodynamic changes is feasible, with the combined evaluation of these two parameters providing incremental diagnostic value for carotid artery stenosis. VFI‐based dual quantitative parameters may offer promising noninvasive diagnostic tools for carotid artery stenosis in high‐stroke‐risk populations.

## Graphical abstract

Evaluation of hemodynamics around atherosclerotic carotid stenosis using turbulence and wall shear stress based on ultrasound vector flow imaging may offer promising noninvasive diagnostic tools for high‐stroke‐risk patients with carotid artery stenosis.

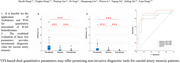

**Abbreviations**
AUC: area under the curve
CTA: computed tomography angiography
DSA: digital subtraction angiography
ICAS: internal carotid artery stenosis
MRI: magnetic resonance imaging
ROC: receiver operating characteristic
ROI: region of interest
Tur: turbulence
VFI: vector flow imaging
WSS: wall shear stress

## 1 Introduction

Ischemic stroke is a prevalent, highly disabling, and deadly disease, ranking as the second leading cause of death worldwide, and carotid artery stenosis accounts for 15%–20% of all cases (Flaherty et al. 2013; Hassani and Fisher 2022). Timely identification and proactive intervention not only enhance patient survival rates but also augment their quality of life, especially among asymptomatic individuals. Additionally, hemodynamics significantly influence the development and progression of atherosclerotic plaques, which in turn impacts the blood flow in the carotid arteries (Huang et al. 2016; Kwak et al. 2014). Therefore, the assessment of hemodynamics in the blood flow surrounding carotid atherosclerotic plaques holds critical importance in the prevention of cardiovascular risks.

Contemporary guidelines prioritize the evaluation of carotid artery stenosis severity as the main determinant in selecting therapeutic strategies. Consequently, a precise diagnosis of carotid artery stenosis holds critical clinical significance. While ultrasound, computed tomography angiography (CTA), magnetic resonance imaging (MRI), and digital subtraction angiography (DSA) are widely used for carotid artery imaging, each has its limitations. DSA, the gold standard for diagnosing stenosis, is invasive and involves radiation (Adla and Adlova 2015; Chilcote et al. 1981). CTA, being noninvasive, requires iodine contrast, which limits its use for patients sensitive to iodine or those with renal insufficiency (Böhm et al. 2017; Bae 2010). MRI, which is sensitive to plaque evaluation, is costly, time‐consuming, and unsuitable for patients with metal implants (Jungmann et al. 2017; Saba et al. 2022). Conversely, ultrasound is the first‐line imaging technique for carotid artery. However, traditional Doppler ultrasound methods, including pulsed wave, continuous wave, and color Doppler, face challenges in intuitively displaying complex flow patterns such as vortices or turbulence (Tur), as well as their specific directional information and angle dependency (Mitchell 1990).

Different from traditional imaging methods, the emerging high‐frame‐rate vector flow imaging (VFI; V Flow) is a novel ultrasound technology that can capture both the magnitude and direction of blood flow velocity (Yiu, Lai, and Yu 2014; Du et al. 2018). This capability allows for the visualization of complex flow patterns, providing detailed visual results of blood flow changes around the carotid artery. Additionally, it can be used to derive various hemodynamic parameters, offering more quantitative information for further clinical research. The Tur index based on high‐frame‐rate VFI enables the quantitative assessment of local blood flow disturbance (Hong et al. 2023). Previous studies have indicated that VFI offers superior precision in hemodynamic assessment compared to conventional Doppler ultrasound (Hong et al. 2023; Goddi et al. 2018). The Tur index enables the quantification of turbulent parameters following arterial stenosis and is associated with complex blood flow patterns (R. Zhao et al. 2022; Dong et al. 2024). Furthermore, the quantitative parameter wall shear stress (WSS) based on VFI refers to the frictional force exerted on the endothelial cell surface during blood flow. Numerous studies have underscored the intimate correlation between WSS and pivotal pathological processes, including atherosclerosis, intimal hyperplasia, plaque genesis, destabilization, and the onset of cerebrovascular events such as stroke (Han et al. 2016; Miura et al. 2013; Carallo et al. 2006; Malek, Alper, and Izumo 1999).

In our previous work, we demonstrated the feasibility of dual‐parameter assessment based on VFI to discern hemodynamics between normal carotid and peripheral arterial flow (Liu et al. 2024; Song et al. 2022). However, the combined quantitative evaluation of moderate/severe carotid stenosis using dual‐parameter assessment remains unexplored. This study aims to quantitatively assess hemodynamic disparities in internal carotid artery stenosis (ICAS) through VFI measurements of Tur index and WSS and to investigate their combined diagnostic efficacy. We hypothesize that the dual quantitative parameters may help to distinguish hemodynamics around stenosis, potentially offering novel diagnostic insights for identifying high‐risk stroke patients.

## 2 Methods

### 2.1 Patients

This prospective study was approved by the Medical Ethics Committee of Shenzhen People's Hospital, and written informed consent was obtained from all participants. A total of 59 patients diagnosed with moderate‐to‐severe carotid artery stenosis were recruited from July 2022 to December 2023. Inclusion criteria: (1) age > 18 years; (2) according to the European Carotid Surgery Trial criteria (Aboyans et al. 2018), the stenosis rate on the B‐mode measurement plane was classified as moderate (50%–69%) or severe (≥ 70%); (3) single internal carotid artery stenosis on one side; and (4) clear B‐mode ultrasound and VFI images obtainable. Exclusion criteria: (1) inability to obtain clear B‐mode ultrasound and VFI images; (2) severe cardiovascular, hepatic, or renal diseases; (3) carotid artery occlusion; (4) history of carotid artery stenting or carotid endarterectomy; and (5) incomplete data cases.

### 2.2 Ultrasound Equipment

All carotid ultrasound examinations were performed by radiologists with over 10 years of experience using the Mindray Resona R9 ultrasound system (Mindray Bio‐Medical Electronics Co., Ltd., Shenzhen, China). The system was equipped with a linear array transducer (L11‐3U) and the updated V Flow imaging function (VFI‐mode).

### 2.3 Data Acquisition of V Flow Imaging

**FIGURE 1:**
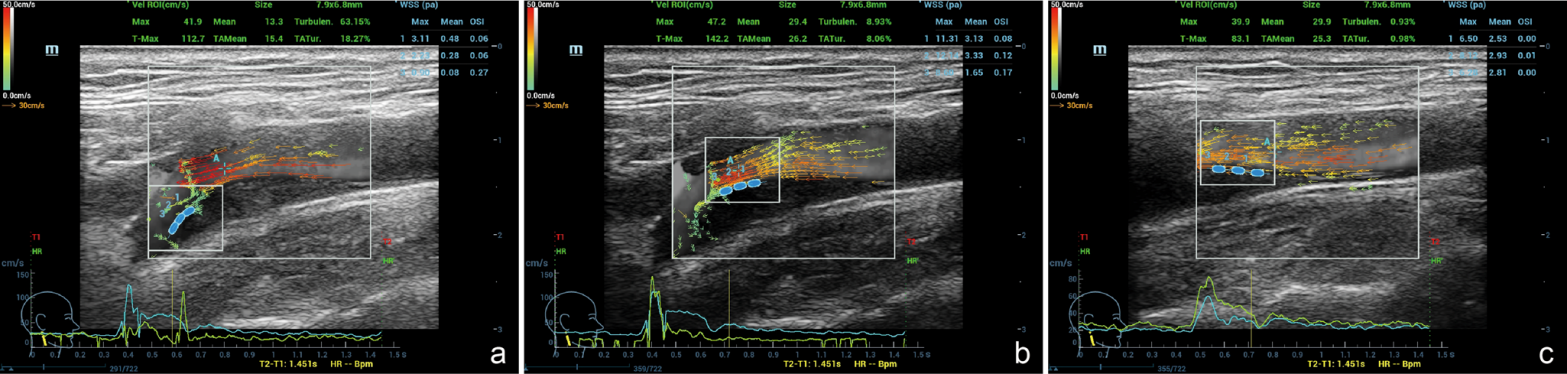
Examples of Tur index and WSS at (a) prestenosis, (b) stenosis site, and (c) poststenosis, respectively. Tur, turbulence; WSS, wall shear stress.

All patients underwent a 15‐min rest period before the examination, followed by lying down with their heads tilted to the contralateral side, exposing the neck. Stenosis degree was measured using grayscale mode, with the maximal luminal diameter at the carotid artery dilation (A) serving as the baseline diameter and the narrowest width at the internal carotid artery (B) as the measurement reference. The stenosis degree was calculated using the formula: stenosis degree = (1 − B/A) × 100%. The prescribed settings for configuring VFI were as outlined: the transducer type was L11‐3U, with a depth set at 3.0 cm. The frame rate (FR) was specified at 498 Hz, while the pulse repetition frequency stood at 4.0 kHz, with an image acquisition duration of 1.5 s. In VFI‐mode, clear images were obtained from three regions: prestenosis, stenosis site, and poststenosis. Dynamic VFI data were acquired by clicking “update” and then saved into the machine for analysis, as illustrated in Figure 1. (1) The “Velocity (region of interest [ROI])” should encompass the regions (prestenosis, stenosis site, and poststenosis) to be measured. (2) Measurement of Tur values: Tur is one of the subfunctions of V Flow and is calculated based on the directions of flow velocities in the “Velocity ROI.” The Tur values are positively correlated with the degree of flow disturbance, ranging from 0 to 1. A value close to or equal to 0 indicates laminar flow in straight vessel, whereas a value nearing 1 represents increased complex blood flow (e.g., turbulent flow or vortex) (Du et al. 2018). Once the designated ROI area for assessment was selected, the machine would automatically calculate and provide the average Tur value, saving the Tur data list predefined within the entire cardiac cycle. (3) Measurement of WSS: aligned the baseline with the vessel wall and repeated the measurement three times at prestenosis, stenosis site, and poststenosis positions. The system would automatically derive the WSS_max_ and WSS_mean_ values within one cardiac cycle. The WSS is also the subfunction of V Flow and is calculated based on the general equation as described (Du et al. 2020).

### 2.4 Statistical Analysis

The mean standard deviation (SD) was used to express continuous variables. Categorical variables were expressed as frequencies and percentages. Tur values and WSS in different stenosis segments were compared using the Kruskal–Wallis test. The relationship of Tur index and WSS against the degree of stenosis was calculated by Spearman's correlation coefficient. Receiver operating characteristic curves (ROCs) were used to compare the diagnostic efficacy of WSS and Tur values in assessing the ICAS. *p* values < 0.05 were considered to indicate statistical significance. All data were calculated using the software programs SPSS Statistics 27, GraphPad Prism 9, and MedCalc.

## 3 Results

### 3.1 Patient Characteristics

The study included a total of 54 patients (5 cases were excluded due to poor image quality), with a mean age of (68.79 ± 11.00) years, including 44 males and 10 females. There were 30 cases of moderate stenosis and 24 cases of severe stenosis. Clinical data of all patients were presented in Table 1.

**TABLE 1:** Clinical characteristics of the participants (*n* = 54).

Characteristics	Mean (SD) or proportion (%, *n*)
Age, y	68.79 (11.00)
Sex, male	81.48% (44)
BMI, kg/m^2^	22.63 (3.16)
Smoking	53.70% (29)
DM	53.70% (29)
CAD	42.59% (23)
SBP, mm Hg	137 (23)
DBP, mm Hg	76 (13)
TC, mmol/L	3.92 (1.24)
TG, mmol/L	1.38 (0.62)
HDL, mmol/L	1.00 (0.23)
LDL, mmol/L	2.32 (0.97)
HCY, umol/L	18.49 (10.3)
GLU, mmol/L	7.03 (3.29)
HbA1c, %	6.95 (1.69)

Abbreviations: BMI, body mass index; CAD, coronary artery disease; DBP, diastolic blood pressure; DM, diabetes mellitus; GLU, glucose; HbA1c, hemoglobinA1c; HCY, homocysteine; HDL, high‐density lipoprotein; LDL, low‐density lipoprotein; SBP, systolic blood pressure; TC, total cholesterol; TG, triglyceride.

### 3.2 Turbulence Index

**FIGURE 2:**
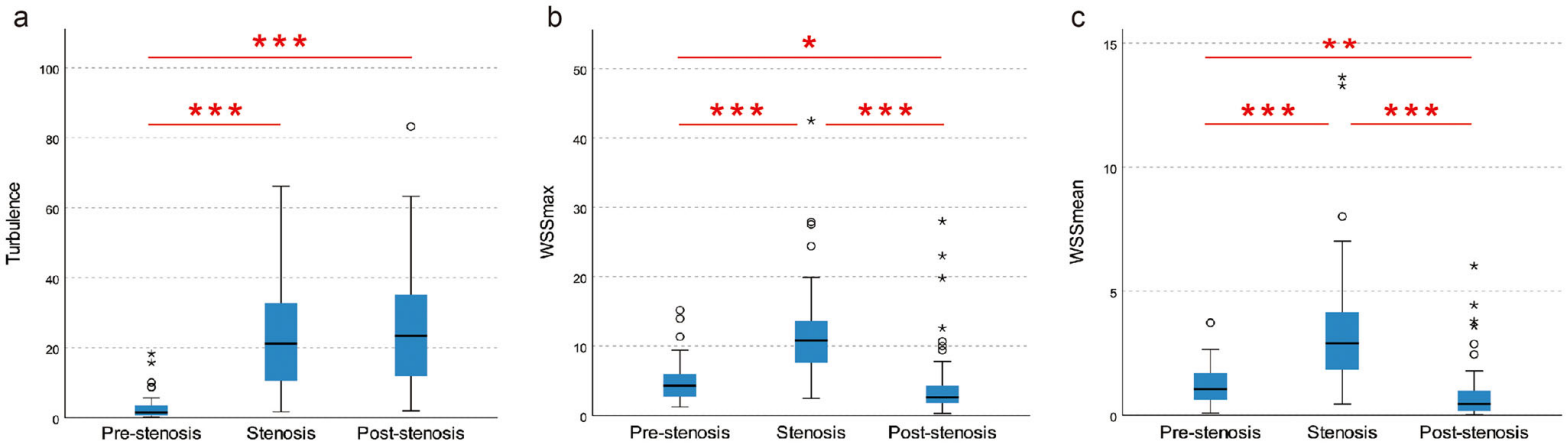
Box plots of (a) Tur index, (b) WSS_max_, and (c) WSS_mean_ for prestenosis, stenosis, and poststenosis segments in patients with ICAS. *p < 0.05, **p < 0.01, ***p < 0.001. ICAS, internal carotid artery stenosis; Tur, turbulence; WSS, wall shear stress.

The average Tur index were (2.88 ± 3.65)% before the stenosis, (23.17 ± 15.52)% at the site of stenosis, and (25.86 ± 17.29)% after the stenosis. Statistical analysis revealed significant differences between prestenosis and stenosis sites, as well as between prestenosis and poststenosis (*p *< 0.001). However, there was no statistically significant difference in Tur values between stenosis sites and poststenosis (*p* > 0.05), as depicted in Table 2 and Figure 2.

**TABLE 2:** Tur index and WSS among different segments around stenosis.

	Prestenosis	Stenosis	Poststenosis	*p* value
Tur index (%)	2.88 ± 3.65	23.17 ± 15.52	25.86 ± 17.29	*p* < 0.01
WSS_max_ (Pa)	4.82 ± 2.87	11.91 ± 6.73	4.43 ± 5.40	*p* < 0.01
WSS_mean_ (Pa)	1.19 ± 0.77	3.42 ± 2.67	0.86 ± 1.21	*p* < 0.01

Abbreviations: Tur, turbulence; WSS, wall shear stress.

### 3.3 WSS Measurement

The WSS_max_ values at the prestenosis, stenosis, and poststenosis regions were (4.82 ± 2.87) Pa, (11.91 ± 6.73) Pa, and (4.43 ± 5.4) Pa, respectively. The corresponding WSS_mean_ values were (1.19 ± 0.77) Pa, (3.42 ± 2.67) Pa, and (0.86 ± 1.21) Pa. Statistical significance was observed for both WSS_max_ and WSS_mean_ values among the three regions mentioned above (*p* < 0.01), as shown in Table 2 and Figure 2.

### 3.4 Comparison of Both WSS_mean_/Tur and WSS_max_/Tur Among Different Segments Around Stenosis

**FIGURE 3:**
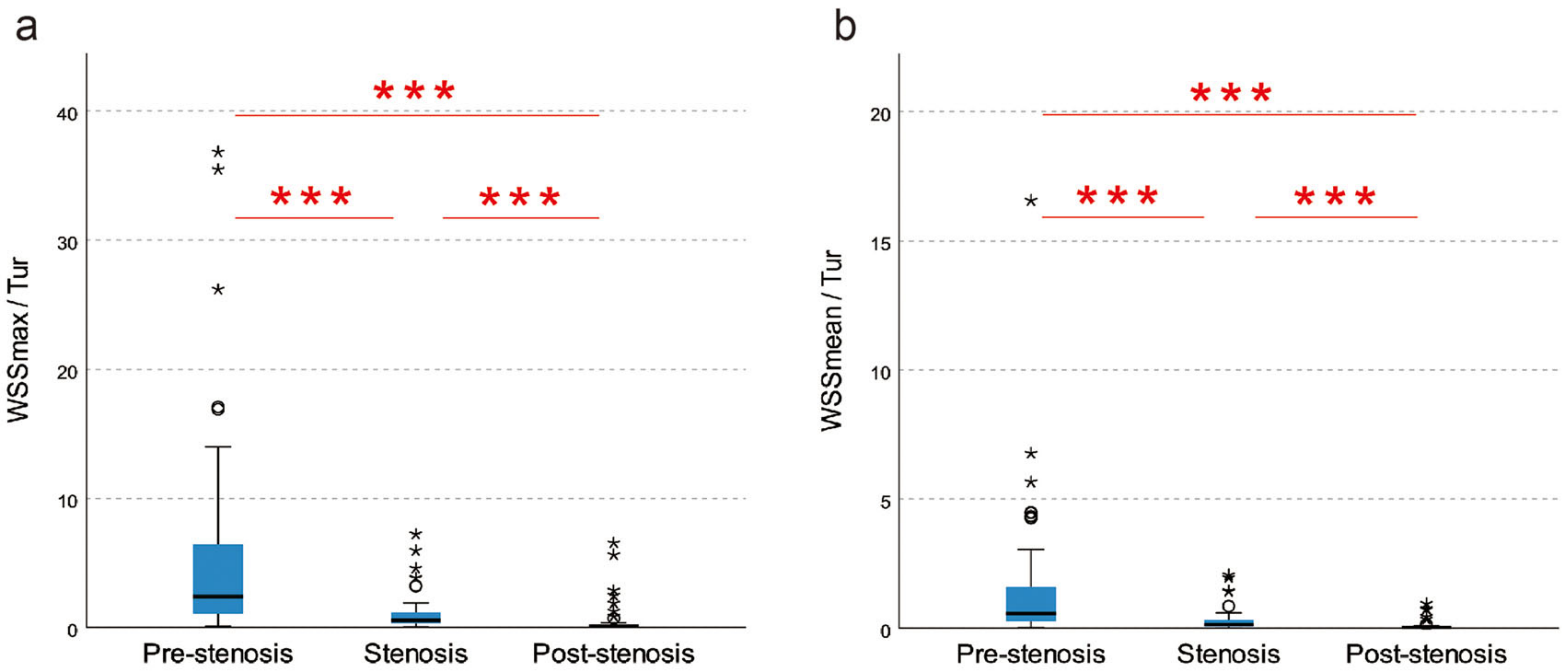
Box plots of (a) WSS_max_/Tur and (b) WSS_mean_/Tur for prestenosis, stenosis, and poststenosis segments in patients with ICAS. ***p < 0.001. ICAS, internal carotid artery stenosis; Tur, turbulence; WSS, wall shear stress.

By defining A as WSS_max_/Tur and B as WSS_mean_/Tur, a comparative analysis was conducted on the A and B values across the prestenosis, stenosis, and poststenosis segments. As shown in Figure 3, A_prestenosis_ > A_stenosis_ > A_poststenosis_ and B_prestenosis_ > B_stenosis_ > B_prestenosis_, all observed differences were found to be statistically significant (*p *< 0.001).

### 3.5 Correlation of Tur index and WSS Against Degree of Stenosis

**FIGURE 4:**
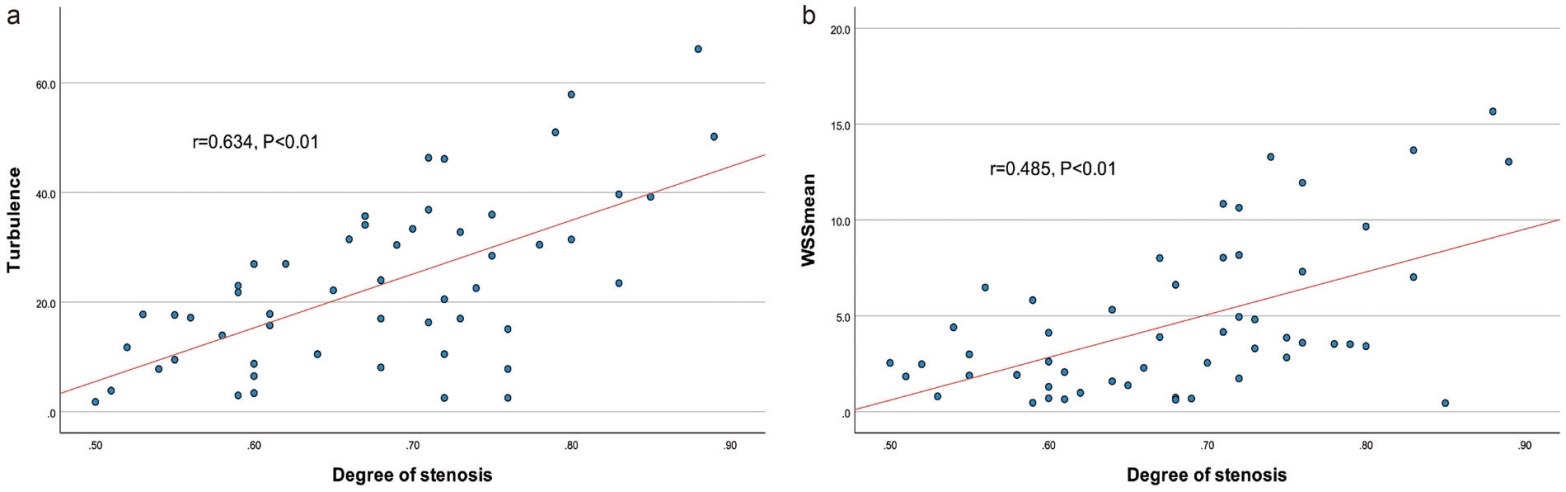
Scatterplots of (a) Tur index and (b) WSS_mean_ against the degree of stenosis. Tur, turbulence; WSS, wall shear stress.

As depicted in Figure 4, the degree of stenosis exhibited a strong positive correlation with Tur index (*r* = 0.634, *p *< 0.001), and a moderate positive correlation with WSS_mean_ (*r* = 0.485, *p* < 0.001).

### 3.6 Diagnostic Value of Tur index and WSS for ICAS

The mean Tur values for moderate and severe stenosis were (17.07 ± 10.07)% and (30.80 ± 17.82)%, respectively. Correspondingly, the mean WSS values were (2.70 ± 2.07) Pa and (4.44 ± 3.26) Pa.

**FIGURE 5:**
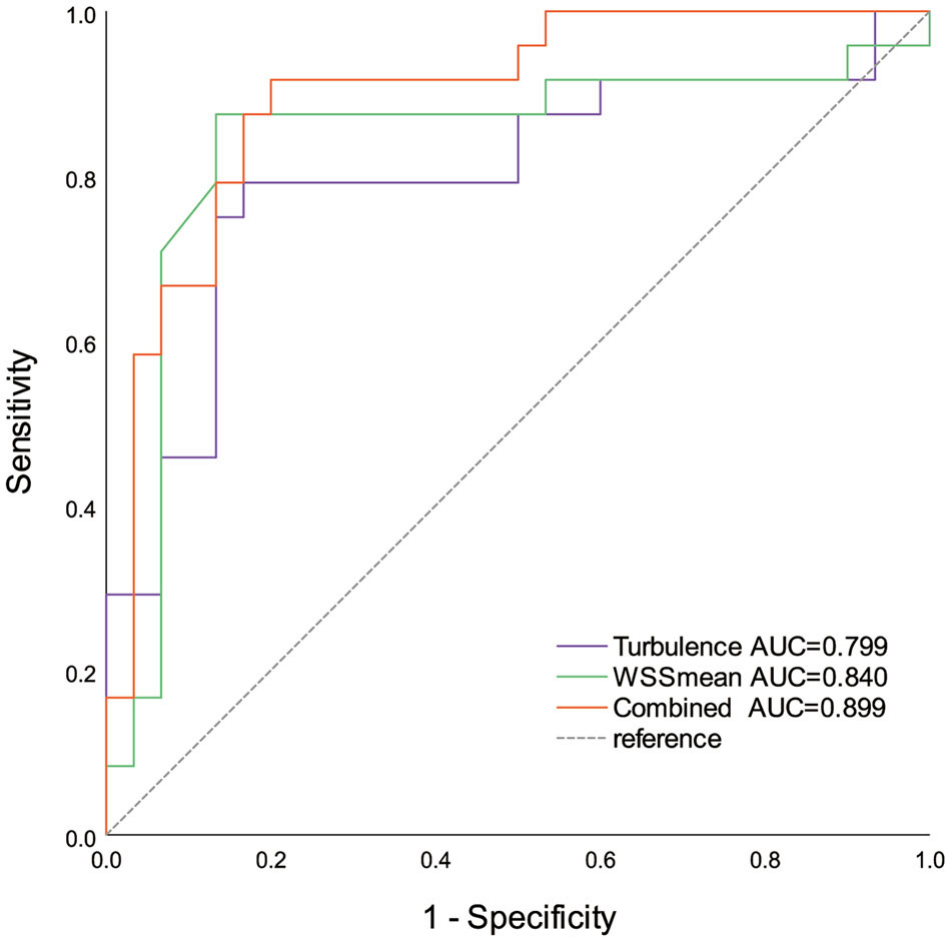
The comparison of diagnostic efficacy of Tur index, WSS, and combined assessment in distinguishing severe carotid atherosclerotic stenosis. Tur, turbulence; WSS, wall shear stress.

An average Tur value exceeding 22.98% yields an AUROC of 0.799 for severe ICAS, with a sensitivity of 79.2% and a specificity of 83.3%. Utilizing an average WSS value greater than 2.647 Pa as a threshold, the AUROC for severe ICAS was 0.840, with a sensitivity of 87.5% and a specificity of 86.7%. The combined diagnostic AUROC (0.899) of Tur index and WSS was significantly higher than that of Tur index or WSS alone, with a sensitivity and specificity of 91.7% and 80.0%, respectively (Figure 5).

## 4 Discussion

Previous studies have confirmed the potential of VFI in assessing carotid atherosclerosis (Gao et al. 2023; Y. Qiu et al. 2020). However, current studies have predominantly focused on single‐parameter evaluations, with limited exploration of dual‐parameter assessments, particularly in populations with moderate‐to‐severe stenosis. To address this gap, our study innovatively integrated Tur index and WSS to quantitatively analyze the hemodynamic characteristics surrounding carotid stenosis. Our findings demonstrated that dual parameters can quantitatively assess hemodynamics in patients with carotid stenosis. Importantly, our results confirmed that the combined use of Tur index and WSS enhanced the diagnostic efficacy of ICAS.

Tur can quantify the degree of blood flow dispersion, yet its clinical significance remains incompletely understood. Our study demonstrated a marked increase in average Tur index at and beyond the stenosis, highlighting significant hemodynamic alterations in these regions, consistent with previous findings (Yiu, Lai, and Yu 2014; Du et al. 2018). An earlier research confirmed a stronger correlation between high Tur values and ulcerated plaques (Dong et al. 2024). R. Zhao et al. (2022) also indicated that as the degree of stenosis increases, Tur values are higher and last longer. In summary, increased Tur values are often accompanied by disrupted blood flow and greater arterial stenosis. Quantitative assessment of Tur index may help us understand the mechanisms of vascular disease formation and progression.

In this study, WSS around different segments of the stenosis showed significant differences. Compared to the prestenosis region, the WSS at the stenosis was markedly elevated, while the WSS poststenosis was significantly reduced. The trend of WSS variation along the plaque is consistent with previous reports (Y. Qiu et al. 2021; Sui et al. 2015; Goudot et al. 2021). It is well known that low WSS or high oscillatory WSS can influence the morphology and function of endothelial cells, closely associated with intimal thickening and the formation of atherosclerosis (Malek, Alper, and Izumo 1999; Hartman et al. 2021). High WSS is generally linked to plaque rupture, while low WSS facilitates plaque development (He et al. 2022; Malik et al. 2022). Consequently, precise measurement of WSS could enhance the identification of patients at high risk for stroke.

Actually, the hemodynamic changes induced by stenosis are quite complex, and there is significant individual variability in carotid blood flow velocity. Therefore, the absolute value of a single parameter may not accurately reflect the trend of hemodynamic changes around the plaque. To overcome the limitations associated with a single parameter, we used the relative ratio of WSS/Tur to study the relative changes in hemodynamic parameters around the stenosis. The results indicated that as important parameters for evaluating atherosclerosis, the combination of WSS and Tur index showed significant regional differences (*p *< 0.001). Previous histopathological studies (Sui et al. 2015) have demonstrated significant differences in the intrinsic components and surface of different regions of plaque stenosis, suggesting that the flow conditions in different parts of the plaque also differ. This finding is consistent with our results.

In this study, the degree of stenosis exhibited a strong positive correlation with Tur index (*r* = 0.634, *p *< 0.001), and a moderate correlation with WSS_mean_ (*r* = 0.485, *p *< 0.001), which is consistent with previous studies (Dong et al. 2024; Y. Qiu et al. 2021; Brandt et al. 2020; Y. J. Qiu et al. 2023). We further compared the area under the curve (AUC) of Tur index and WSS in diagnosing carotid artery stenosis. The results indicated that the combined diagnostic AUROC (0.899) of Tur index and WSS was significantly higher than that of Tur index (AUC = 0.799) or WSS (AUC = 0.840) alone. Those results suggest VFI‐based dual quantitative parameters may offer promising noninvasive diagnostic tools for carotid artery stenosis patients. Previous studies (Matz et al. 2017; Cassola et al. 2022) have demonstrated that ultrasound, CTA, and MRA provide comparable results in the diagnosis of internal carotid artery stenosis. de Athayde Soares et al. (2024) indicated that ultrasound, as the exclusive preoperative imaging modality for carotid assessment, yields postoperative outcomes similar to those derived from preoperative evaluations with CTA/MRA. Our findings further validated that the incorporation of dual quantitative parameters significantly enhanced the diagnostic utility of ultrasound as a non‐invasive preoperative assessment imaging technique.

In previous research, R. Zhao et al. (2022) used Tur index to assess turbulent flow changes in the distal segment of the canine femoral artery with varying stenosis degrees. Dong et al. (2024) assessed hemodynamic disparities in different stenotic artery locations with Tur. Y. J. Qiu et al. (2023) investigated the correlation between symptomatic stenotic arteries and WSS, all above relying on single parameters. However, single‐parameter assessments may lack objectivity and fail to fully capture hemodynamic changes induced by stenosis. While other researchers (Liu et al. 2024; Song et al. 2022; M. Zhao et al. 2024) explored Tur index and WSS correlations in carotid and peripheral vessels, their studies were confined to healthy or mildly stenotic populations, leaving the relationship between Tur index and WSS in moderate/severe stenotic arteries and adjacent regions unclear. Therefore, our study is the first to integrate Tur index and WSS in investigating moderate‐to‐severe stenosis populations. Our results further validate the feasibility of dual‐parameter quantitative assessment of hemodynamics in moderate‐to‐severe stenosis, offering potential novel non‐invasive diagnostic tools for high‐stroke‐risk patients.

## 5 Limitations

First, the sample size in our study was relatively small, and only patients with moderate‐to‐severe stenosis were included. There was a lack of hemodynamic parameter comparison with patients who had mild stenosis. In future research, we plan to increase the sample size to further validate the diagnostic value of V Flow in carotid artery stenosis. Second, VFI is currently a two‐dimensional imaging technique, which limits the accuracy of WSS and Tur index measurements to some extent. The development of three‐dimensional VFI imaging technology will help us accurately detect blood flow information outside the imaging plane. Finally, the relationship between hemodynamics and stroke risk is quite complex. This study did not conduct long‐term follow‐up of the subjects. Future prospective studies are needed to assess the impact of hemodynamic changes on ischemic stroke.

## 6 Conclusion

The Tur index and WSS at and around the stenotic region exhibited significant variability, using a single parameter to predict the progression of the lesion might provide misleading information. This study is the first to explore the feasibility of combining Tur index and WSS for the quantitative assessment of hemodynamic characteristics in patients with moderate‐to‐severe carotid artery stenosis. The combined use of these two parameters added incremental value to the diagnosis of carotid artery stenosis. In conclusion, VFI is a simple and practical quantitative imaging method for carotid artery stenosis. The dual quantitative parameters, Tur index and WSS, are expected to become new noninvasive diagnostic tools for identifying high‐stroke‐risk patients with carotid artery stenosis.

## Author Contributions

**Shaofu Hong**: methodology, writing–original draft, investigation. **Yinghui Dong**: writing–original draft, methodology. **Wenjing Gao**: data curation, investigation. **Di Song**: data curation, investigation. **Mengmeng Liu**: data curation, investigation. **Weiyue Li**: formal analysis, resources. **Yigang Du**: resources, methodology. **Jinfeng Xu**: writing–review and editing, project administration, supervision. **Fajin Dong**: writing–review and editing, project administration, funding acquisition, supervision.

## Conflicts of Interest

Yigang Du is employed by Mindray. The other authors declare no conflicts of interest.

### Peer Review

The peer review history for this article is available at https://publons.com/publon/10.1002/brb3.70150

## Data Availability

The data that support the findings of this study are available on request from the corresponding author. The data are not publicly available due to privacy or ethical restrictions.
